# UnSafengine64: A Safengine Unpacker for 64-Bit Windows Environments and Detailed Analysis Results on Safengine 2.4.0

**DOI:** 10.3390/s24030840

**Published:** 2024-01-27

**Authors:** Seokwoo Choi, Taejoo Chang, Yongsu Park

**Affiliations:** 1 The Affiliated Institute of ETRI, P.O. Box 1, Yuseong, Daejeon 305-600, Republic of Korea; seogu.choi@gmail.com (S.C.); sobawoo@gmail.com (T.C.); 2 Department of Computer Science, Hanyang University, Wangshimriro 222, Seongdonggu, Seoul 04763, Republic of Korea

**Keywords:** anti-forensics, code obfuscation, dynamic code analysis, software reverse engineering, computer security

## Abstract

Despite recent remarkable advances in binary code analysis, malware developers still use complex anti-reversing techniques that make analysis difficult. Packers are used to protect malware, which are (commercial) tools that contain diverse anti-reversing techniques, including code encryption, anti-debugging, and code virtualization. In this study, we present UnSafengine64: a Safengine unpacker for 64-bit Windows. UnSafengine64 can correctly unpack packed executables using Safengine, which is considered one of the most complex commercial packers in Windows environments; to the best of our knowledge, there have been no published analysis results. UnSafengine64 was developed as a plug-in for Pin, which is one of the most widely used dynamic analysis tools for Microsoft Windows. In addition, we utilized Detect It Easy (DIE), IDA Pro, x64Dbg, and x64Unpack as auxiliary tools for deep analysis. Using UnSafengine64, we can analyze obfuscated calls for major application programming interface (API) functions or conduct fine-grained analyses at the instruction level. Furthermore, UnSafengine64 detects anti-debugging code chunks, captures a memory dump of the target process, and unpacks packed files. To verify the effectiveness of our scheme, experiments were conducted using Safengine 2.4.0. The experimental results show that UnSafengine64 correctly executes packed executable files and successfully produces an unpacked version. Based on this, we provided detailed analysis results for the obfuscated executable file generated using Safengine 2.4.0.

## 1. Introduction

Recently, malware has increased as a potential vulnerability for industrial control systems with sensors and actuators. For example, it was reported that BlackEnergy 3 malware is under suspicion of having caused the massive blackout at the Ukraine Power Grid [1].

Malware developers use various code obfuscation techniques to deter code analysis and protect their copyrights. Modern (commercial) obfuscation tools, including VMProtect [2], Safengine [3], and Themida [4], employ strong anti-reverse engineering techniques and are actively utilized for hindering analysis [5].

Although each has a different behavior, these protection tools use a common technique called code packing, which compresses or encrypts a target program for protection. Specifically, it transforms a target program into a packed program by compressing or encrypting the code into packed data and associating it with an unpacking routine. Additionally, these tools include various anti-reverse engineering techniques (for example, anti-debugging [5], self-modifying code [6], and code encryption [6]) in the unpacking routine, making analysis difficult.

To the best of our knowledge, Safengine [3] is considered one of the most complex commercial protectors in Microsoft Windows environments and there have been no analysis results published. Safengine is a code-packing tool that encrypts a target executable. To deter analysis, Safengine contains diverse anti-reversing techniques, including anti-debugging, anti-dump, anti-trace, integrity checking, and API relocation [3].

To analyze packed software, static analysis, which analyzes without execution, is limited because the target code is encrypted or compressed. To overcome this problem, dynamic analysis, which conducts code execution and analysis simultaneously, can be used. To analyze dynamically, debuggers [7] or dynamic binary instrumentation (DBI) tools [8,9] are widely used; however, they have shortcomings in that the execution environments are slightly different from those of the actual execution, and many anti-reverse engineering techniques can detect this difference.

In this study, we present UnSafengine64, a Safengine unpacker for 64-bit Windows environments. UnSafengine64 relies on Pin [9], one of the most accurate DBI tools for Windows. We used Detect It Easy (DIE) [10], IDA Pro [11], x64Unpack [12], and x64Dbg [13] as auxiliary analysis tools.

UnSafengine64 can automatically decode the obfuscated application program interface (API) function calls using the algorithm presented in [14]. Moreover, UnSafengine64 provides diverse functionalities for analyzing packed software, including automatic identification of the original entry point (OEP: the entry point of the original target program; refer to Section 2.1) [15,16], detecting anti-debugging routines, dumping the memory region, and automatically unpacking the packed file. The complete source code for UnSafengine64 is available in [17].

To verify the effectiveness of the proposed scheme, we conducted experiments on Safengine 2.4.0 in 64-bit Microsoft Windows. UnSafengine64 successfully unpacks all the test files packed with this protector and produces detailed logs. Based on this, we explain in detail the structure of the obfuscated files generated using Safengine 2.4.0. 

The remainder of this paper is organized as follows: Section 2 deals with preliminaries (Section 2.1), an overview of Pin (Section 2.2), and related works (Section 2.3). In Section 3, UnSafengine64, the proposed automatic unpacker for Safengine, is described. Section 4 summarizes the experimental results, and Section 5 provides detailed analysis results of the unpacking routine of Safengine 2.4.0. Finally, we conclude the paper in Section 6.

## 2. Preliminaries and Related Work

Section 2.1 shows a simplified unpacking procedure for packers, including Safen-gine. In Section 2.2, we briefly provide an overview of Pin, which UnSafengine64 relies on. Section 2.3 describes related work.

### 2.1. (Very Simplified) Unpacking Procedure of Packers

**Figure 1:**
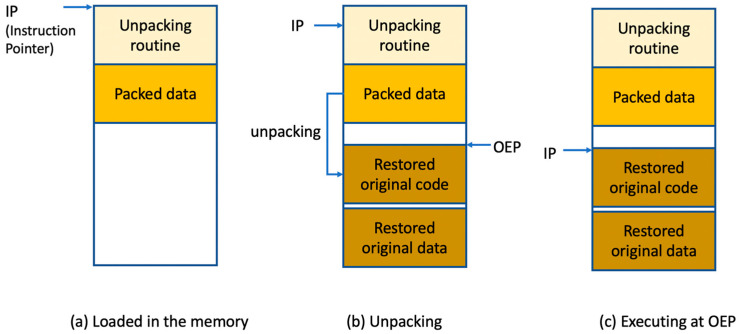
Very simplified unpacking procedure of packers (including Safengine).

Code packing is the process of transforming a target program into a packed program such that it compresses or encrypts the original code and associates the packed code with the unpacking routine. Figure 1 shows the general unpacking procedure when a packed program is run. After the packed program begins (Figure 1a), the execution flow proceeds to the restoration routine, which we call the unpacking routine. It unpacks or decrypts packed data and restores the original code and data. When this work is completed (Figure 1b), the unpacking routine also restores the execution context of the original program, which includes the initialization of the CPU registers. Subsequently, as shown in Figure 1c), it sets the program counter to the entry point of the unpacked original code, where we call this address OEP. Finally, the restored original code is used. In reality, the unpacking procedure of Safengine is very complex; it uses code packing multiple times while using various anti-reversing techniques to deter the analysis.

### 2.2. Overview of Pin [9]

Pin [9] is one of the most widely used DBI tools for Windows and Linux environments. Pin was originally used for computer architecture analysis; however, it is now actively used for diverse purposes, such as checking code coverage for optimization, finding memory leaks, and analyzing security properties. 

Pin performs binary instrumentation; it instruments executables at runtime using a just-in-time (JIT) compiler. It works as follows: (1) Pin intercepts the execution of the first machine instruction for the executable target. (2) For the basic block (code for one entry point and only one exit), starting with this instruction, Pin compiles a new code. (3) It executes this new code, which is almost identical to the original code; however, Pin ensures that it retains control when a branch exits this basic block. (4) After regaining control, Pin compiles more code for the branch target and continues execution. 

Pin allows us to inject our own plug-in code (instrumentation) called Pintool. Pintool consists of the following two elements:

- A mechanism for deciding insertion points where the plug-in code is inserted;
- Plug-in code for execution at the insertion points.

These two elements are the instrumentation and analysis codes. Using Pintool, we can execute the target binary code and our plug-in code for analysis. For example, we can write a simple plug-in code in which the analysis code increments the counter by one, and we insert this analysis code between every executed machine instruction of the target program. After executing the target program, the counter has the number of all executed instructions.

The potential limitation of Pin is that artifacts arise when executing Pin. Therefore, malware can detect the presence of Pin. If the unpacking routine detects Pin and aborts the execution, further analysis cannot be performed. (If a detection routine is found, the code can be patched to neutralize the detection. However, this is not the fundamental solution.).

### 2.3. Related Work

Relevant research has been actively conducted on malware analysis (including malware detection [18,19], malware removal [20], and ransomware detection [21]) and binary code analysis [22,23]. However, anti-reversing techniques have not attracted special interest from researchers except for specific topics such as code virtualization [24,25]. This section summarizes the related research and tools for unpacking, which can be classified into three categories: debuggers, DBI, and (specialized) unpacking schemes or tools. The details of each topic are as follows.

Debugger: Because the debugger supports runtime disassembly for the target program and the single-step execution of machine instructions, it is one of the most convenient and widely used tools for the dynamic analysis of binary code (including malware). In a debugging environment, the execution context can be monitored and modified promptly. Debugging tools allow analysts to easily check how the execution status changes as each machine instruction is executed. However, the debugging environment differs slightly from the actual execution environment, and anti-debugging techniques can detect this difference.

WinDbg is a debugger developed by Microsoft and has the advantage of being able to debug kernels. However, WinDbg has the shortcoming of a relatively inconvenient interface compared to other debuggers. OllyDbg [7] and immunity debuggers are the most widely used debuggers for analyzing binary code in Microsoft Windows environments; however, they support only 32-bit binaries. x64Dbg [13] is one of the most widely used debuggers for 64-bit binaries. 

Recently, Apate [26] was proposed by Hao Shi and Jelena Mirkovic as a framework for hiding the debugger from anti-debugging techniques. The authors designed and implemented diverse bypassing methods for anti-debugging techniques in 32-bit Windows. Apate was implemented as a WinDbg plug-in program. The authors claimed that Apate outperforms other debugger-hiding schemes by a wide margin [26]. In our experiment, although Apate worked well on simple packers (ASPack or UPX), it failed on complex packers, including Themida, VMProtect, and Safengine. 

Dynamic binary instrumentation (DBI): For dynamic analysis, DBI is widely used because it can execute an analyst’s code while the target code is being run. DBI operates in such a way that the target and analyst codes are interleaved and executed. Pin [9] provides various convenient API functions for analyzing the target code and is known to be one of the best tools for correct execution and fastness. However, it is difficult to identify the cause of an error because it is not open-source.

Valgrind [27] and DynamoRIO [9] are widely used because they are open-source and provide convenient API functions for analyzing binary code. Valgrind only supports the Linux operating system, whereas DynamoRIO often fails to analyze a large/complex program. Detours [28] hooks the major Win32 API functions for analysis and is well known for its efficiency and correctness. However, one drawback is that a detailed analysis (instruction-level analysis) is difficult.

These DBIs focus on fast and accurate code execution and flexible code instrumentation for target binaries. Because the original code is modified during code instrumentation, anti-reversing techniques for detecting this modification cannot be avoided. As there are few types of DBI tools, techniques for identifying individual DBIs have already been widely used [29,30]. For example, Lee et al. [31] presented the detailed analysis results of anti-VM and anti-DBI techniques on commercial protectors: Themida, VMProtect, Obsidium, and ASProtect. In addition, they demonstrated that their bypassing work was successful in Pin environments. 

Unpacking schemes/tools: Unpacking tools have been developed for various packers. One such example is the UPX unpacker [32]. Renovo [15] provided a general method to determine the unpacking procedures. It identifies the write-and-execute behaviors that are common in unpacking. However, Renovo does not deal with various anti-reverse engineering techniques, making it less practical in the real world.

VMAttack [33] focused on the automatic de-obfuscation (intensive code stripping) of code-virtualization techniques in VMProtect (version 2), whereas this study deals with the automatic analysis of the unpacking procedure of Safengine.

Pindemonium [34], one of the most well-known open-source unpacking tools, relies on Pin for analyzing the packed program and dumping the unpacked code. To the best of our knowledge, it cannot unpack the recent versions of complex (commercial) packers, including Safengine. 

Choi et al. proposed HybridEmu [35], which is a dynamic analysis scheme for investigating the internal structure of malicious code in Microsoft Windows 32-bit environments. Similar to xUnpack64 [12], HybridEmu can directly call or emulate various API functions in malware while emulating instructions using a 32-bit CPU simulator. However, it was designed only for 32-bit environments. 

x64Unpack [12] is a hybrid dynamic analysis tool for coping with diverse packers in 64-bit Windows environments; it can handle both finding general unpacking routines and evading various anti-reverse engineering techniques. In addition, Choi et al. [12] provided detailed analysis results for VMProtect. In this study, we used x64Unpack to obtain the analysis results for Safengine.

Recently, UnThemida [36] was developed as a plug-in for the Pin tool to analyze and unpack the structure of files packed with Themida 2.4.5.

One of the fundamental approaches to cope with secure computation is using quantum cryptography. It can directly offer information-theoretic security against possible attacks from obfuscated malware [37,38].

## 3. UnSafengine64: Safengine Unpacker for 64-Bit Windows Environments

Section 3.1 describes an overview of UnSafengine64. We explain the details of UnSafengine64 in Section 3.2, Section 3.3, Section 3.4, Section 3.5, Section 3.6, Section 3.7, Section 3.8, Section 3.9, Section 3.10 and Section 3.11.

### 3.1. Overview of UnSafengine64

**Figure 2:**
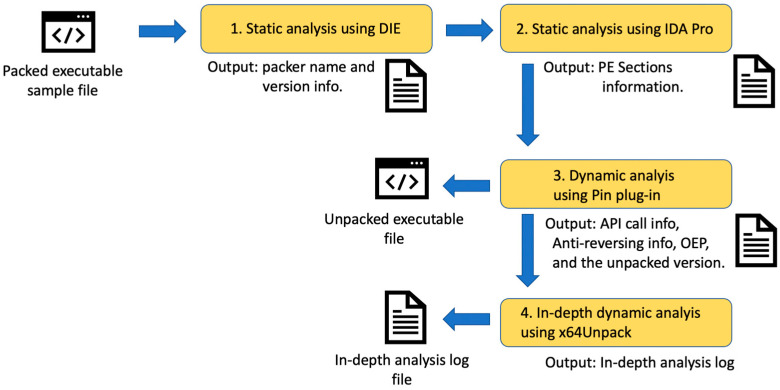
Overall execution procedure of UnSafengine64.

UnSafengine64 was developed as a plug-in for Pin [9]. We chose Pin because it is one of the most accurate dynamic analysis tools for Windows. Our plug-in program can analyze the unpacking routine while bypassing the anti-reverse engineering techniques of Safengine. With this plug-in, we can analyze Safengine’s internal structure, detect OEP, and perform automatic unpacking. In addition, relevant information can be gathered to detect anti-debugging code, dump memory, and restore obfuscated files. We also used the following auxiliary tools for a deep analysis:

- Detect It Easy [10]: DIE identifies the type of packer in the executable file and outputs the section information. DIE was used to determine whether the target program was packed with Safengine.
- IDA Pro [11]: IDA Pro is one of the most popular disassemblers for reverse engineering binaries in Windows/Linux environments. The executable file is statically disassembled and displayed, and diverse information, including the function structure and section information, is displayed. Because some malware uses Safengine’s unique signature to cheat DIE, IDA Pro can be used for double-checking in this case.
- x64Dbg [13]: x64Dbg is a widely used debugger for reversing 64-bit Windows environments. We used x64Dbg as an auxiliary tool for executing code chunks to double-check the analysis results from Pin or x64Unpack. In addition, x64Dbg can be used to determine whether UnSafengine64 is properly unpacked. Sometimes, the unpacked version does not execute because of a minor bug; therefore, we can use x64Dbg to fix the minor bugs.
- x64Unpack [12]: This is an application-level hybrid emulator that either directly executes code chunks or emulates them. Using x64Unpack, we can monitor API function calls, examine memory reads/writes, and emulate each instruction for detailed analysis. For further information, refer to [12]. We used x64Unpack to analyze the major functionalities of Safengine, as explained in Section 4. For unpacking, x64Unpack is not required (Figure 2).

The analysis process for UnSafengine64 proceeds as follows: First, we prepared a sample program packed with Safengine. The first step was to conduct a simple static analysis using DIE [10]. DIE displays the section and packer information. In this step, we confirmed that the target binary was packed with Safengine.

The second step was a static analysis using IDA Pro. IDA Pro cannot precisely analyze packed binary code because the original code is compressed or encrypted and stored as data. We used IDA Pro to obtain the section information and Import Address Table (IAT) information. After obtaining the unpacked version in the final step, IDA Pro was used to check whether the unpacked version was correctly built. 

The third step was to conduct a dynamic analysis using the Pin plug-in program UnSafengine64. It executes target code chunks while monitoring threads, analyzing obfuscated API function calls, and detecting or bypassing anti-reversing routines. The OEP can also be determined in this step. Finally, the unpacked program is automatically dumped.

Optionally, we can proceed to the fifth step, which involves a dynamic analysis using x64Unpack. The x64Unpack emulator can also correctly emulate obfuscated files, analyze unpacked code chunks, and determine the OEP.

In addition, we optionally used x64Dbg as an auxiliary tool. Because x64Dbg does not properly execute the unpacking routine (owing to various anti-reversing techniques), we used x64Dbg as an auxiliary tool to double-check whether a specific functionality was correctly analyzed.

Among these, DIE, IDA Pro, and x64Dbg are widely used tools; however, their detailed descriptions are beyond the scope of this study. x64Unpack is described in detail in [12]. Hereafter, we focus on our Pin plug-in, UnSafengine64.

### 3.2. Structure of UnSafengine64 

**Figure 3:**
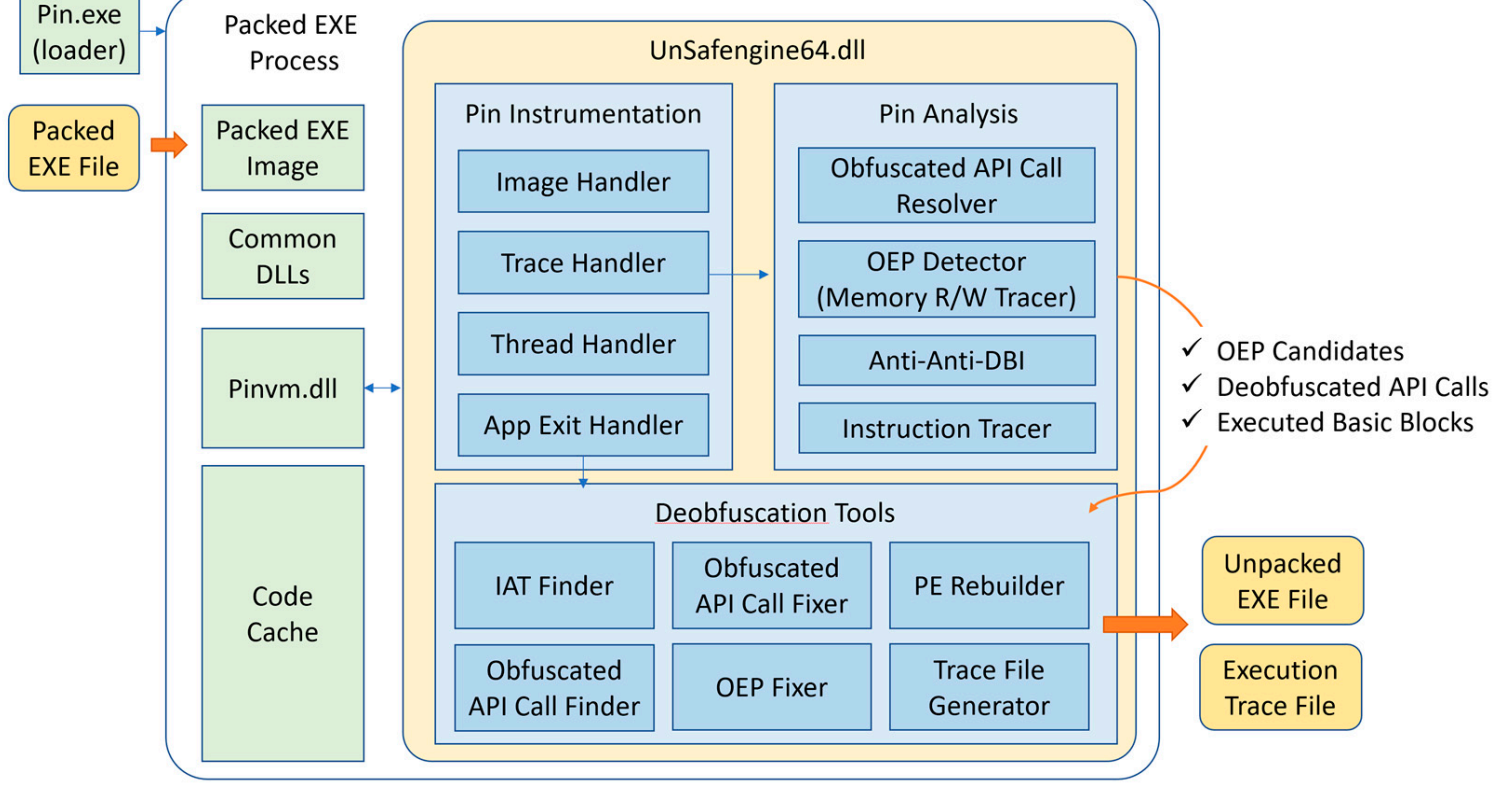
Internal structure of UnSafengine64.

UnSafengine64 functions as follows: First, Pin creates a new process in a suspended state that contains the executable code of the target (already packed with Safengine). Pin loads pinvm.dll to use Pin API functions in the process and SafengineAnalyzer.dll, which is the file name of our Pin plugin tool (UnSafengine64), into memory. Subsequently, it starts the execution (Figure 3).

UnSafengine64 consists of four components for instrumentation: the image handler, trace handler, thread handler, and application exit handler. The image handler handles the application/library binary image, and the trace handler handles the basic blocks in the code cache of Pin. The thread handler tracks the start and end of each thread. The application exit handler detects the OEP at the end of the unpacking procedure. This is explained in Section 3.3, Section 3.4, Section 3.5 and Section 3.6.

For the analysis, UnSafengine64 has four components: an Obfuscated API call resolver, an OEP detector, an anti-DBI, and an instruction tracer, as explained in Section 3.7, Section 3.8, Section 3.9 and Section 3.10. We address the implementation issues in Section 3.11.

### 3.3. Pin Plug-In Structure: Image Handler 

The image handler is invoked when the target executable image files/DLL library files are loaded. It stores the addresses of the loaded executable files and the loaded DLL files. In addition, it stores the names and the start/end addresses of the functions in them. This information is used to identify which part we are currently executing the code in.

### 3.4. Pin Plug-In Structure: Trace Handler

The trace handler is called when there is no JIT-translated code (by Pin) in the code cache. We define the instruction trace as the sequence of executed instruction information, specifically the sequence of the basic blocks. The main task of the trace handler is instrumentation to execute the analysis routines, such as instruction tracers and OEP detectors, during the execution of the packed file. In addition, obfuscated API function calls are analyzed using an obfuscated API call resolver during unpacking.

Memory access must be tracked to detect OEP and anti-debugging code chunks. Because the unpacking routine with anti-reversing techniques accesses the PEB (Process Environment Block) or TEB (Thread Environment Block), the memory accesses for these special areas of memory must be tracked. For OEP detection, to use the heuristic algorithm (which will be described in Section 3.7), the memory writes and execution accesses should be tracked and recorded. 

### 3.5. Pin Plug-In Structure: Thread Handler

The thread handler maintains the logs of the start and end of the thread. This information is then used by the trace handler to enable instruction tracing for each thread.

### 3.6. Pin Plug-In Structure: Application Exit Handler

When UnSafengine64 detects an OEP (described in Section 3.7), it calls the application exit handler. It de-obfuscates the obfuscated API function calls and restores the code near the OEP, which is modified by the OEP obfuscation technique from Safengine. It also reconstructs the PE (Portable Executable) structure modified by Safengine’s anti-dump functionality. In Pin environments, Safengine occasionally stops when I/O occurs frequently, and we use a delayed-logging approach such that writing the log into the file is delayed until the unpacked file is created. Before the OEP is met, UnSafengine64 stores all log information in memory.

### 3.7. Pin Plug-In Structure: OEP Detector

We suppose that the target program was packed using Safengine. Recall that OEP is the address of the beginning point of the original code, which is restored and loaded into memory during unpacking.

Our algorithm for finding OEP is based on [15]: The OEP detector finds the “write-and-execute” case, where the execution (instruction pointer) branches to the memory region that has been written after the start-up. If found, the OEP detector regards the address as an OEP candidate. Generally, it finds a large number of OEP candidates because Safengine uses self-modifying code. Therefore, we used an algorithm [16] to refine the OEP candidates.

If this approach is used, the obtained OEP is slightly different from the actual OEP. This is because Safengine uses the OEP obfuscation technique. We identified a specific pattern (set of instructions) for Safengine’s OEP address. Therefore, the OEP detector performs pattern matching to fix the OEP address.

### 3.8. Pin Plug-In Structure: De-Obfuscating Function Calls

Many commercial protectors use diverse API obfuscation techniques to hinder analysis; the three major ones [14] are as follows:

- Obfuscation of call/jmp instructions that go to the beginning of the API functions (the first method);
- Obfuscation for the Import Address Table (IAT) (the second method);
- Concealing either a portion or the entirety of the code of the API function body through obfuscation (the third method).

**Figure 4:**
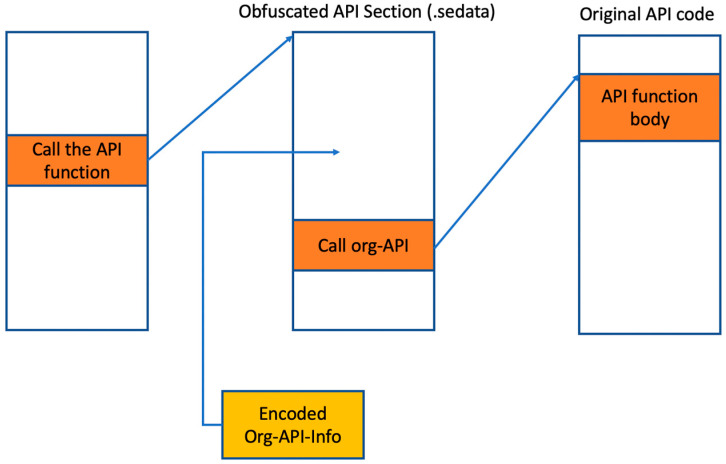
Obfuscated API function calls in Safengine.

Among these, Safengine uses only the first. In Safengine, each call/jmp instruction for the API function call is changed to several instructions with the same meaning (the first method). In this case, jmp or call crosses different sections, as shown in Figure 4.

In the figure, “Call the API function” implies the instruction for calling the API function. “Obfuscated API Section ()” has the obfuscation-related instructions, which are added by Safengine. This section presents several branch instructions for code obfuscation. “Encoded Org-API-Info” indicates the encoded or encrypted address of the API function body, where the original API function address is encoded or encrypted. If there is a call to an obfuscated API function, this value is decoded or decrypted and used to jump to the beginning of the original API function. 

To de-obfuscate a function call is as follows: In Safengine, the control flow for an obfuscated API function calling eventually leads to an API function body in the DLL regions. To identify the original API function address, the control flow of all candidates for the obfuscated API call is first determined. Subsequently, the control flow for each candidate is executed for verification. The correct flow is identified by checking whether the API function in the DLL has executed. This method is called the run-until-API method [14] and can also be used to de-obfuscate API function calls in other commercial protectors, including Themida or VMProtect.

The peculiarity of Safengine’s API obfuscation is that once the address of the obfuscated API function is computed, it jumps quickly to the computed address without repeating the same process. When the first obfuscated API function call is executed, the starting address of the API function body is calculated and stored in memory, and then the body is called. Second, it checks whether the function address calculation has already been performed, and then calls the corresponding address.

To analyze this, we designed and implemented an obfuscated-API-function call resolver. In the first execution, this de-obfuscation tool saves the current execution context at the end of execution. Then, in the second execution, at the OEP, it stops execution, performs the de-obfuscation of API function calls using the context information, and then produces the de-obfuscated execution file.

We used the algorithm in [14] to check whether the last address of each trace was in the section containing the code (added by Safengine) for calling obfuscated API functions or in the DLL region (containing the API function body). Sometimes, the analyzed last address is an invalid address, such that it causes an execution error (this is because static analysis using the disassembler is not always perfect, since Safengine uses self-modification while it puts garbage bytes between the instructions). In this case, we simply ruled out the addresses. 

Occasionally, Safengine calls GetModuleHandleA() when calling the obfuscated API functions, and we should handle this case by tracking the call and return of GetModuleHandleA(). 

### 3.9. Pin Plug-In Structure: Anti-Anti-DBI

In our experience, generally, 64-bit protectors do not contain as many anti-reversing techniques as 32-bit packers. However, Safengine uses various anti-reversing techniques for deterrent analysis (explained in Section 5), hardware breakpoint detection, software breakpoint detection, the detection of various debuggers, the detection of virtual machine environments, and checking file integrity. Pin can automatically bypass many of these. Here, we describe a single-step detection technique in which a vanilla Pin is detected. 

The single step is an anti-reversing technique that can be used in both 32-bit and 64-bit Windows. This method causes Pin to quit abnormally. In this technique, to set the trap flag (TF: 0x100) of the RFLAGS register, the Safengine unpacker first places a specific value on the stack to set the trap flag and then executes the POPFQ instruction. During normal execution, the correct exception handler is executed. If Pin is executed, exception handling is not performed, and the program is abnormally terminated. 

Whenever UnSafengine64 executes a POPFQ instruction, it checks the value at the top of the stack to determine whether the TF is set. If the TF is set, it goes manually to the exception handler. In the case of a PE32+ (64-bit PE) file, because the exception handler is specified in the PE header, UnSafengine64 parses the PE header in advance and jumps to the corresponding exception handler when executing the file. 

### 3.10. Pin Plug-In Structure: Instruction Tracer

The instruction tracer has trace-logging and unpacking functionalities. If the trace logging immediately writes the log into the log file every time an instruction is executed, just as in a logging program such as Intel Pin SDE (Software Development Emulator), execution will be very slow, and the execution is aborted via anti-reverse engineering techniques that measure the execution time. Therefore, we store all the execution traces in memory and write them to the file at the time when the program exits. 

Safengine uses a large amount of memory because it executes at least 1.3 billion instructions, even in a simple sample program. When we log the instruction trace, approximately 27 GB of memory is used to execute the sample program up to the OEP. The size of the text file resulting from the trace was approximately 17 GB, even when only the addresses of the basic blocks and names of the executed API functions were recorded. Minimizing the log data is left for future work. 

### 3.11. Pin Plug-In Structure: Implementation Issues

The proposed Pin plug-in tool was implemented using Visual Studio 2019 and Pin 3.18 in Windows 11 64-bit Version 22H2. Because previously published anti-DBI schemes or papers [6,15,16] rely on old versions of Pin, it is difficult to use them in the recent Windows environment.

The main difficulty in implementing the Pin tool for analyzing obfuscated code is that the execution trace is large, and it is difficult to analyze the cause when the execution aborts, owing to anti-reversing techniques. 

UnSafengine64 has a CUI interface for outputting analysis results, dumping memory, or producing an unpacked executable file. Furthermore, the user can set a specific memory region to trace the instruction execution for deep analysis. 

## 4. Experimental Results

To verify the effectiveness of the proposed scheme, we first prepared Protector Safengine 2.4.0 [3]. We then developed a test program. It calls a few simple API functions related to the message box and strings. That is, the program generates a pseudo-random number, outputs it in the message box, and exits. We compiled this program using Visual Studio 2019 in the release mode with default options.

**Figure 5:**
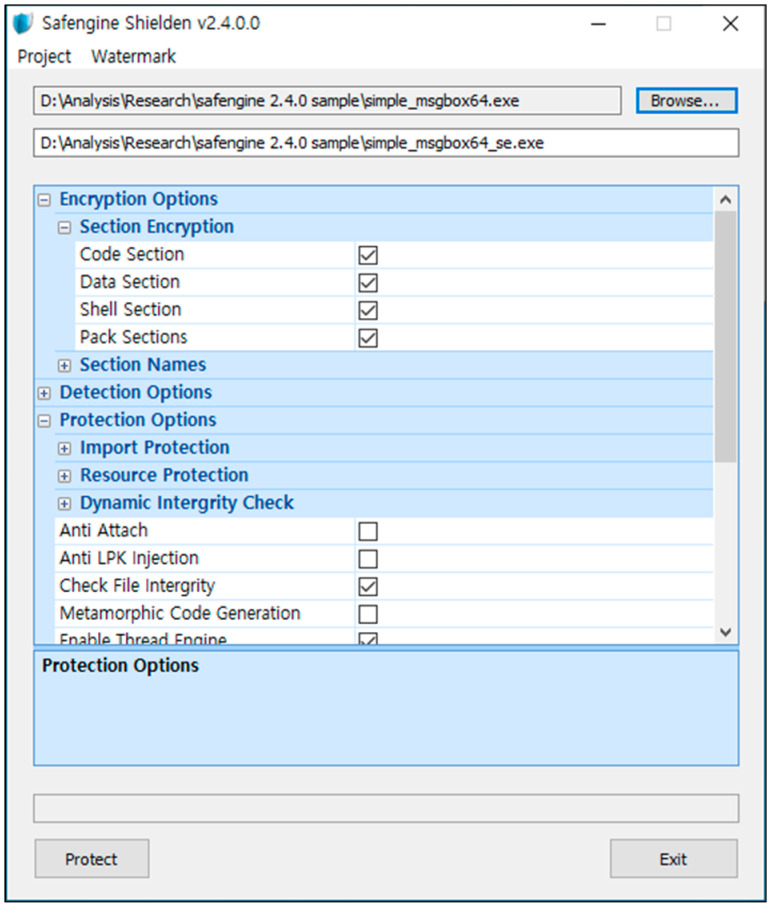
Screenshot of Safengine 2.4.0.0 packer.

For packing, we used the following options (as shown in Figure 5): DetectSoftICE: enabled; Detect Syser: enabled; Detect OllyDbg: enabled; Detect E Language Dumpers: enabled; Detect FileMon: enabled; Detect RegMon: enabled; Detect Virtualization Tools: enabled; Detect Debugging Events: enabled; Eliminate IAT: enabled; Resource Encryption: enabled; Check File Integrity: enabled; and Enable Thread Engine: enabled. 

**Figure 6:**
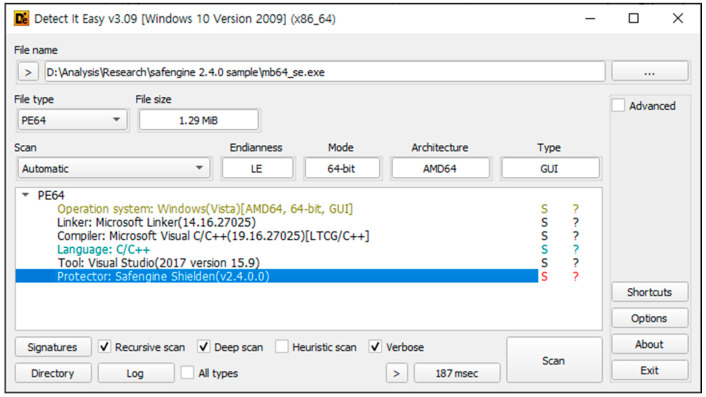
Analysis results of DIE 3.09.

The first step of the analysis was a static analysis using DIE 3.09. This confirmed that the target program was packed with Safengine 2. In addition, it shows that the original program was compiled using Visual Studio 2019 (shown in Figure 6). 

**Figure 7:**
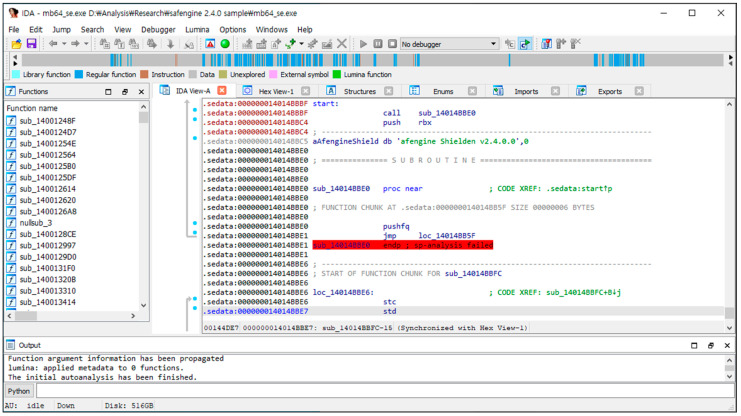
Screenshot of IDA Pro for the packed program.

The second step was a static analysis using IDA Pro. IDA Pro cannot properly analyze the packed code, which indicates that the original compressed program appears as data. However, using IDA Pro, we can obtain the section information, which is explained in Section 5 (Figure 7).

**Figure 8:**
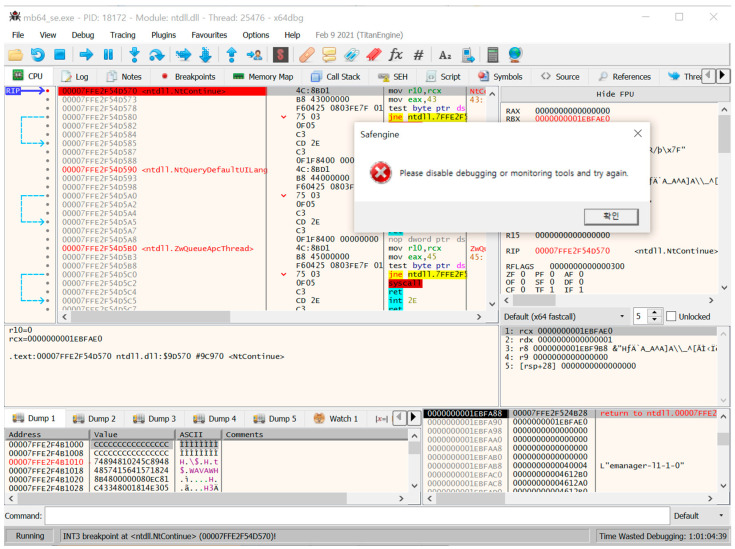
Screenshot of x64Dbg for the packed program.

The third step involves a dynamic analysis using x64Dbg. Because x64Dbg does not properly execute the unpacking routine (owing to various anti-reversing techniques), we used x64Dbg as an auxiliary tool to check whether the suspected part of a specific debugger detection technique was working correctly (Figure 8).

The fourth step was a dynamic analysis using the Pin plug-in program, UnSafengine64. When we ran a vanilla Pin that did not have any plug-ins, we observed that an internal error appeared and stopped. We confirmed that our plug-in tool, UnSafengine64, successfully executed the target program and outputted the unpacked code. In addition, it produced log messages regarding the API function calls and information on the executed basic blocks and threads.

**Figure 9:**
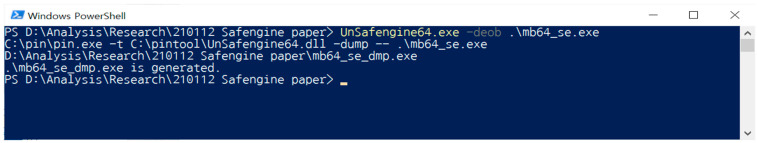
Screenshot of UnSafengine64 execution.

We confirmed that approximately 10^12^ instructions were executed. As shown in Figure 9, after execution, we obtained an unpacked version of the sample program.

**Figure 10:**
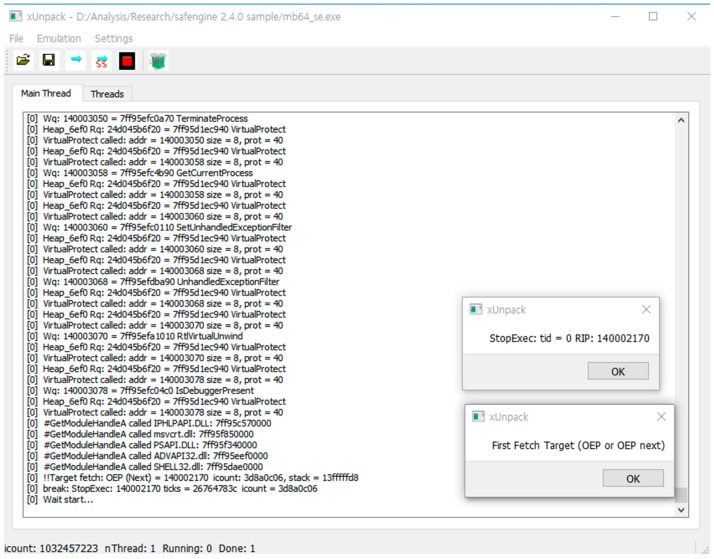
Screenshot of x64Unpack for the packed program.

The fifth step was a dynamic analysis using x64Unpack. x64Unpack also successfully executed the target program and output the trace log, as shown in Figure 10.

In summary, we confirmed that UnSafengine64 successfully bypasses anti-reverse engineering techniques, stops at the OEP, and generates an unpacked executable file. We confirmed that the unpacked file could be disassembled and decomposed using IDA Pro. In addition, we confirmed that it can be dynamically analyzed using an x64Dbg debugger without being disturbed by anti-reversing techniques.

## 5. Detailed Analysis Results of the Packed Files Using Safengine 2.4.0

This section explains the analysis results for an executable packed using Safengine 2.4.0. First, we obtained the trace information using UnSafengine64 and then analyzed the trace information; for example, we tried to identify what kind of anti-reverse engineering techniques are used and at which execution point the compressed code is uncompressed.

Section 5.1 compares the section structures of the original and packed versions of the executable files. Section 5.2 explains the obfuscation technique of API function calls, and Section 5.3 describes the Import Address Table (IAT) of the packed file. Section 5.4 explains the unpacking procedure for executing obfuscated files.

### 5.1. Comparison Results on the Sections in the PE File

**Figure 11:**
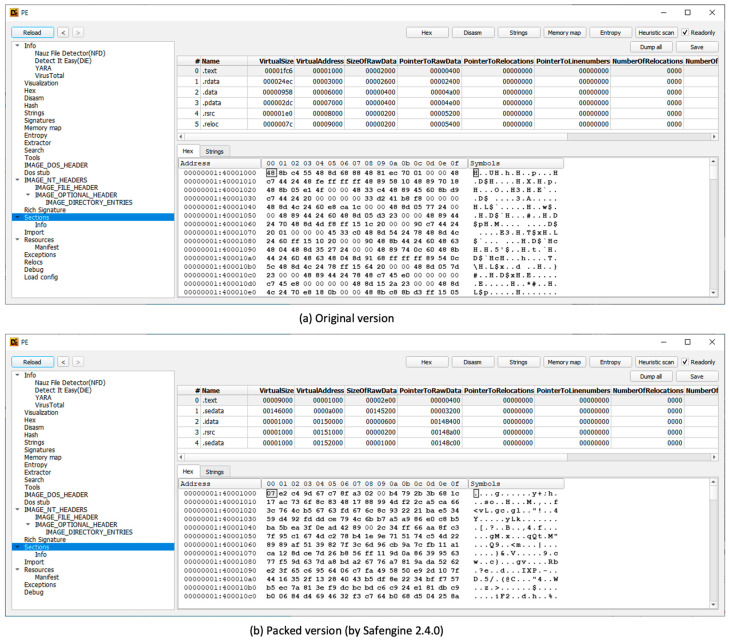
The section structure of the original executable file and the packed version.

For the original and packed files, we compared the section information as follows: Figure 11 shows the section information for the original file and packed version (Safengine). Safengine obfuscates the original file and merges it into one section: the “.text”, “.rdata”, “.data”, “.pdata”, and “.rsrc” sections of the original executable file correspond to the “.text” section of the packed files. A virtual address range of the “.reloc” section is included in the “.text” section of the packed file and “.reloc” section data were deleted when the original file was packed. The “.sedata” section contains the code necessary for the unpacking process, and the remaining sections contain the necessary content for the unpacking process. 

### 5.2. Analysis of the API Function Obfuscation

**Figure 12:**
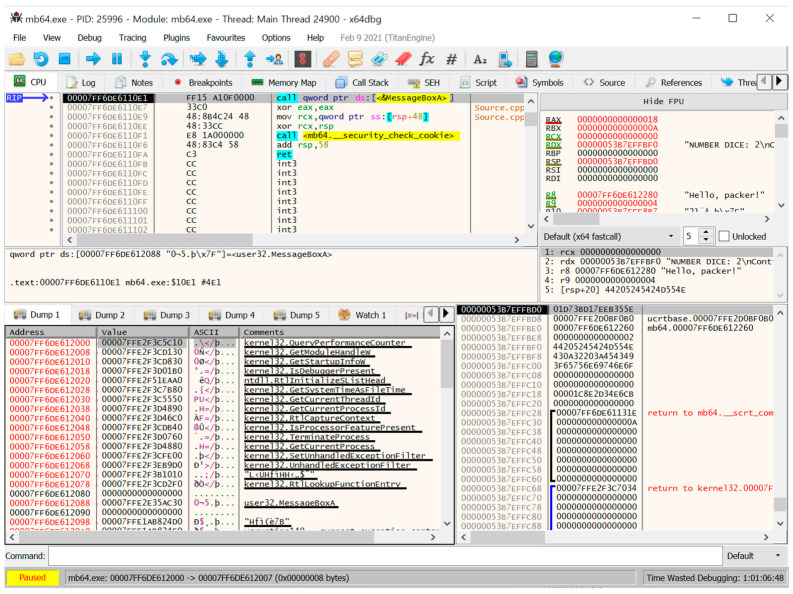
The original instruction for calling MessageBoxA().

In general, (commercial) protectors use diverse API functional obfuscation techniques to deter analysis on API function calls. We explain the API function obfuscation of Safengine by showing an example, that is, how the “call MessageBoxA()” instruction is obfuscated in Safengine. Figure 12 shows the original instructions for calling MessageBoxA(). The first line shows a function call for MessageBoxA().

**Figure 13:**
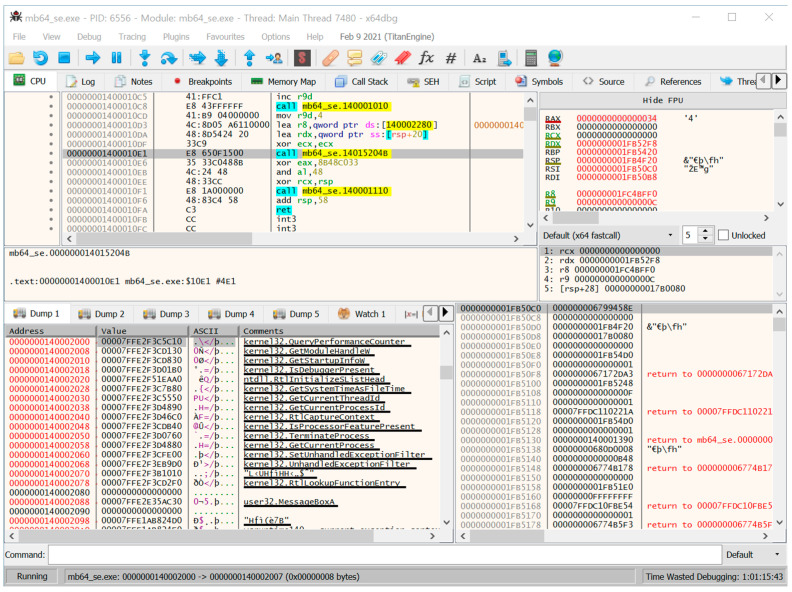
The obfuscated instructions for calling MessageBoxA().

Figure 13 shows the obfuscated instructions for calling MessageBoxA(). This screenshot was obtained using a simple plug-in tool for Pin, which stops in the OEP and then attaches the debugger, x64Dbg, to the suspended process. We observed that the target address of the call function is changed to 0x14015204b. In the original program, the next instruction of “call MessageBoxA()” is “xor eax, eax”, whereas the next instruction of the obfuscated call, “call 0x14015204b”, is “xor eax, 0x8b48c033”. This disassembly failure in the debugger is because Safengine intentionally placed a random value, which is “0x35” in this case, after the call instruction. When returning after the call, it comes to 0x1400010e7; therefore, “xor eax, eax” is executed normally. 

**Figure 14:**
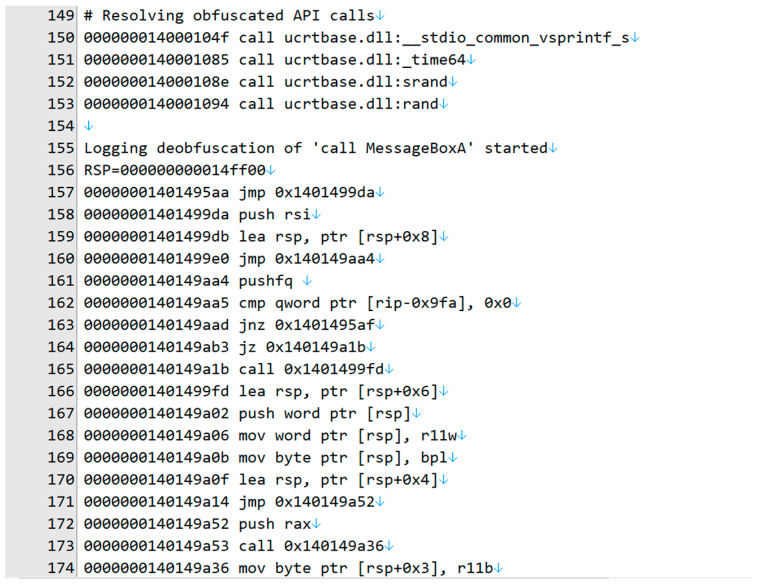
The instruction trace for calling MessageBoxA(): part 1.

Traces of the de-obfuscation procedure were recorded using UnSafengine64. First, the obfuscated call goes to 0x14014955aa. Then, the instruction “cmp qword ptr [rip-0x9fa], 0x0” that compares [rip-0x9fa] with 0 at address 0x140149aa5 is executed. [rip-0x9fa] is a disassembly type provided by the disassembly library of Pin, and its original address is 0x1401490b3. The restored value is stored in the address after the API function address has been restored (Figure 14).

**Figure 15:**
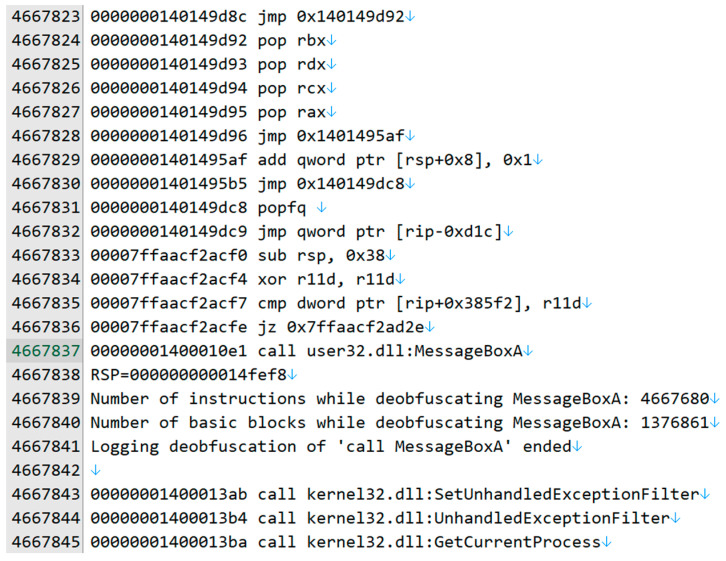
The instruction trace for calling MessageBoxA(): part 2.

The last instruction at 0x140149dc9 in the “.sedata” section is “jmp qword ptr [rip-0xd1c]”, which is “jmp qword ptr [0x1401490b3].” The memory buffer at 0x1401490b3, which is the same address as the cmp instruction at 0x140149aa5, holds the de-obfuscated MessageBoxA() address. After executing 1,376,861 basic blocks and 4,667,680 instructions, the body of MessageBoxA() was executed (Figure 15).

Finally, the RSP register values are adjusted. In this case, because the RSP increased by eight during obfuscation, the RSP was adjusted to restore the stack top position when the call instruction was executed in the original program. 

### 5.3. IAT (Import Address Table) of the Packed File

The following figure shows the structure of the IAT when unpacking is completed and the original program is executed (packed version). The original version indicates the address and functional names of each item in the IAT of the original executable file. When we compare the original and packed versions, the relative addresses of the IAT are consistent, implying that Safengine preserves some or all the addresses in the IAT area after unpacking. 

When comparing the original and packed versions using Safengine, we can observe that the IAT points to the same function. However, when executed, Safengine does not read and use the IAT but directly calls the obfuscated API functions. (The Safengine developer explains that the IAT is reserved for compatibility.) 

**Figure 16:**
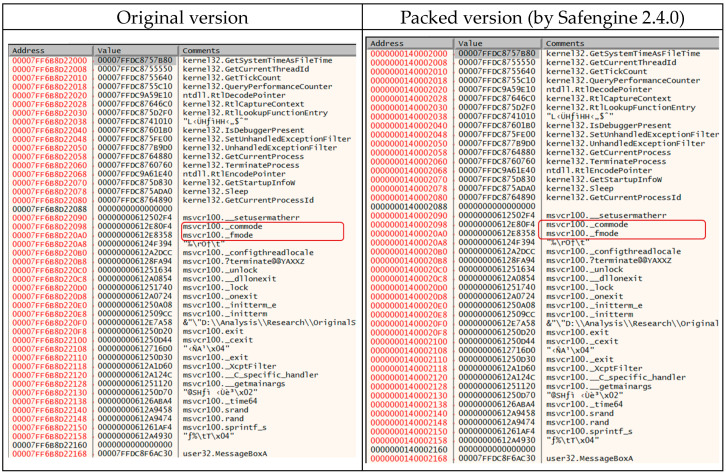
IAT (Import Address Table) of the original executable file and the packed version.

The red boxed _fmode and _commode shown in Figure 16 are the API data variables, which are used as global variables internally by the Microsoft CRT (C-RunTime) library. These global variables in the IAT are not accessed at runtime because they are obfuscated by Safengine. (Themida and VMProtect do not obfuscate API variables and use data variables to access the IAT.).

### 5.4. Analysis Results of the Unpacking Procedure (Safengine 2.4.0)

When we execute an obfuscated executable file packed with Safengine 2.4.0, the unpacking routine conducts the following work until the OEP is met (after which the code of the original file is executed). These functionalities are arranged in the order of execution. 

**① Obtaining the handle for kernelbase.dll:**

First, the unpacking routine retrieves the handle of the second KernelBase.dll from the InitializationOrderModuleList of the PEB’s _PEB_LDR_DATA structure. 

**② Restoring API function addresses:**

Unlike other obfuscation tools, Safengine executes many instructions at the beginning (approximately 800 million instructions are executed in this case). These instructions recover the addresses of the API functions used in Safengine’s unpacking routines. In Safengine, function addresses are encoded or encrypted by default. Therefore, Safengine’s unpacking routine calls the API function after decrypting the encoded API function address instead of calling it directly. 

**③ Calling TlsAlloc**() **to allocate memory buffers:**

Packers sometimes use local thread storage (TLS) to avoid analyses. The TLS memory area allocated in Safengine is used for the checksum. To detect hardware breakpoints, the unpacking routine first stores the relevant register context in the TLS and then compares the stored value with the current register context. If they differ, then a hardware breakpoint exists. A detailed explanation is provided in ⑧. 

**④ Deleting resource directory entries;**

Next, Safengine deletes the resource directory entries. It is difficult to guess exactly why this was carried out, but we assume that it is likely to hide sensitive data from being analyzed in the resource area when a memory dump is performed.

**⑤ CRT** (**C-RunTime Library**) **initialization function call:**

This calls the initialization functions related to the CRT (C-RunTime Library). The unpacking routine initializes these to use the C-RunTime Library functions or DLL (e.g., MSVCRT.DLL) during the unpacking procedure.

**⑥ Detecting and disabling debuggers:**

The unpacking routine detects debuggers and attempts to block them using the technique described in [5]. When calling the NtSetInformationThread() function, the second parameter (THREADINFOCLASS ThreadInformationClass) is as follows: it passes the 0x11 value to the ThreadInformationClass; 0x11 is undocumented, which indicates ThreadHideFromDebugger. When this function is called, an event is sent to the kernel, such that the debugger is no longer attached.

**⑦ Changing section attributes to disable software breakpoints:**

Software breakpoint is the technique that debuggers generally use for debugging, and it works as follows: If we overwrite INT 3 (=0xcc) to the specific instruction in the desired position, when this instruction is executed, a software interrupt occurs when the corresponding position is executed. The debugger catches this interrupt, restores the original code, and continues execution.

If we change the section attributes of the memory to disable write access, we can prevent software breakpoints because the instructions cannot be changed. 

**⑧ Setting to detect changes in hardware breakpoints:**

To detect a hardware breakpoint, it sets the hardware breakpoint values to arbitrary values, calculates the checksum, and stores it in the TLS buffer. Subsequently, the watchdog threads compare the stored checksum values and current registers (DR[0]–DR[5]) to check for the presence of hardware breakpoints. This process was also performed on all watchdog threads.

We can see the execution trace that sets DR[0], DR[1], DR[2], and DR[3] registers. Safengine computes the checksum as follows: In this case, 0x1c022 = (0x140007016 + 0x140007000 + 0x140007004 + 0x140007008); mod 232 becomes the checksum value. Later, if this value is recalculated and is different, the hardware breakpoint value is regarded as changed, and the execution aborts.

**⑨ Checking file integrity:**

The unpacking routine reads the executable file and checks its integrity. To achieve this, it calls GetMappedFileNameW() to obtain a file handle. It then retrieves the file name using the QueryDosDeviceW() function. This creates a file path with GetLogicalDriveStringW() with this file name. Using the CreateFileW(), GetFileSize(), and ReadFile() functions, this file is loaded into memory, the hash value is calculated, and its integrity is checked by comparing it with the hash value (already entered by Safengine) in the executable file.

**⑩ Detecting debugger tools:**

First, it checks whether the \\.\NTICE driver is loaded or not to detect the SOFTICE debugger. It then checks for the presence of the SYSERBOOT device driver to detect the Syser debugger.

The unpacking routine patches the DbgBreakPoint() and DbgUserBreakPoint() functions to prevent breakpoints. This modifies the first byte of the DbgBreakPoint() and DbgUserBreakPoint() function bodies; in this case, 0xeb. In the 80x86 environment, 0xeb implies a jump instruction. These functions are called when the debugger is attached; however, if we change the values in this manner, the debugger cannot be attached; that is, it jumps to an arbitrary address. The reason for calling VirtualProtect() is to obtain write access.

**⑪ Watchdog thread creation:**

This creates eight watchdog threads to detect whether they are being debugged or not.

**⑫ Checking system manufacturer registry keys for virtual environment detection:**

The system manufacturer registry key is used to detect the virtual environments. For example, for VMWare, the system manufacturer string is “VMware, Inc. (Palo Alto, CA, USA)” VirtualBox contains the “VBOX” string. Therefore, if these values are included in the Windows Registry, the unpacking routine is executed in a virtual environment. In addition, if there is a word such as VMWare or VBOX, it also looks for the BIOS key and is regarded as running in a virtual environment.

We tested in the VirtualBox environment and confirmed that the virtual environment is detected by checking this registry key.

**⑬ Saving error message strings:**

This allocates a memory buffer and stores the error message strings. The error strings are encrypted.

**⑭ Decoding the target code:**

This process decrypts the original compressed or encrypted code. First, the compressed or encrypted data are copied to the buffer. Then, it deletes the original data. Subsequently, the compressed data are decompressed. Finally, the encrypted data are decrypted.

**⑮ Restoring IAT** (**Import Address Table**) **settings:**

As the default option of Safengine, API obfuscation is performed: Safengine deletes the IAT table, writes the API address in the middle of the PE image, and calls the API function indirectly using it. It then restores the IAT table during Safengine’s unpacking procedure.

**⑯ OEP execution:**

Safengine executes instructions at the OEP. Subsequently, the original target code is executed.

## 6. Conclusions

In this study, we propose a Safengine unpacker for 64-bit Windows Environments: UnSafengine64. UnSafengine64 was developed as a plug-in for Pin. To verify the effectiveness of our scheme, experiments were conducted using Safengine 2.4.0. The experimental results show that UnSafengine64 correctly executes packed executable files and successfully produces an unpacked version. Based on this, we provided detailed analysis results for the obfuscated executable file generated using Safengine 2.4.0. The complete source code for UnSafengine64 is available in [17]. Because UnSafengine64 currently cannot run a 32-bit version of Safengine, future work will include the support of 32-bit programs and other commercial packers.

## Data Availability

No new data were created or analyzed in this study. Data sharing is not applicable to this article.
